# Novel Insights Into Epigenetic Reprogramming and Destabilization of Pericentromeric Heterochromatin in Cancer

**DOI:** 10.3389/fonc.2020.594163

**Published:** 2020-11-05

**Authors:** Morten Frier Gjerstorff

**Affiliations:** ^1^Department of Cancer and Inflammation Research, Institute for Molecular Medicine, University of Southern Denmark, Odense, Denmark; ^2^Department of Oncology, Odense University Hospital, Odense, Denmark; ^3^Academy of Geriatric Cancer Research (AgeCare), Odense University Hospital, Odense, Denmark

**Keywords:** cancer, genomic instability, chromosome 1q12, pericentromeric heterochromatin, polycomb group proteins, synovial sarcoma, breakpoint X, genomic amplification

## Abstract

Pericentromeric heterochromatin is maintained in a condensed structure by repressive epigenetic control mechanisms and perturbation of these may cause diseases. The chromosome 1q12 region harbors the largest pericentromeric heterochromatin domain in the genome and is among the most common breakpoints in both solid and hematopoietic cancers. Furthermore, the 1q arm is frequently amplified in cancer and this may support tumorigenesis by increasing the dosage of the many oncogenes of this genomic region. Recent studies have provided insight into the mechanisms leading to loss of 1q12 stability and 1q amplification and DNA hypomethylation seems to play a prominent role. This may be the result of decreased activity of DNA methyltransferases and instrumental for 1q12 destabilization or arise secondary to perturbation of other important epigenetic mechanisms that control repression of pericentromeric heterochromatin. Polycomb proteins were recently demonstrated to epigenetically reprogram demethylated 1q12 pericentromeric heterochromatin in premalignant and malignant cells to form large subnuclear structures known as polycomb bodies. This may influence the regulation and stability of 1q12 pericentromeric heterochromatin and/or the distribution of polycomb factors to support tumorigenesis. This review will discuss recent insight into the epigenetic perturbations causing the destabilization of 1q12 pericentromeric heterochromatin and its possible implications for tumor biology.

## Introduction

Loss of genomic stability is an enabling feature of tumor progression, in which elevated rates of mutations and numerical/structural chromosomal deviations drive the development of cancer hallmarks (1). Generally, there are three different types of genomic instability: base pair mutation, microsatellite instability and chromosome instability (2). The latter describes events associated with mitotic missegregation that lead to changes in chromosome number and chromosome rearrangements that produce abnormal chromosome structure. Such chromosomal rearrangements may be non-random involving specific parts of the genome and multiple studies have implicated pericentromeric/juxtacentromeric heterochromatin (PCH) in this type of genomic instability. The largest PCH domain in the genome is located at the chromosome 1q arm and comprises a megabase stretch of satellite II and III DNA repeats. Similar structures are present at other chromosomes such as 9 and 10, but these are smaller and have different satellite DNA compositions. The cellular functions of PCH still remain largely elusive, but these gene poor regions appear to support centromere function in mitosis and be essential for architectural and topological organization of the nuclear department (3–5). Importantly, the 1q PCH seems to play a prominent role in tumorigenesis.

## Chromosome 1q12 Breakage and 1q Amplification in Solid Cancers

This 1q PCH domain is among the most frequent breakpoint sites in cancer (6, 7). For instance, the most frequent karyotypic aberration in breast cancer involves 1q PCH, leading to isochromosomal formation, translocation (often to 16p) or less often deletion of the whole 1q arm (8). This may in some cases be the only karyotypic change in breast cancer tumors, suggesting a role in tumorigenesis (9). In melanoma, the 1q arm is frequently amplified with about 25% of cutaneous primary tumors and metastases exhibiting 1q copy number gain (10) and was found to correlate with the transition from melanoma in-situ to invasive lesions (11). The presence of 1q12 aberrations in melanoma was confirmed by another study, where as many as 90% of chromosome 1 rearrangements occurred in the 1q12 region confirming the importance of chromosome 1q PCH in chromosome 1 instability (12). In this line, copy number gain at chromosome 1q is also among the most frequent genomic alterations in hepatocellular carcinoma (13–16) and in some cases this again involves the 1q12 region (17, 18). Strikingly, 1q amplifications are also highly frequent in a number of pediatric solid cancers, including tumors of the CNS (19, 20) and kidney (21, 22).

## Chromosome 1q12 Breakage and 1q Amplification in Hematopoietic Cancers

In hematopoietic cancers, gain of chromosome 1q is also one of the most common cytogenetic aberrations. It is very well described in multiple myeloma (MM) where up to 40% of patients with abnormal karyotypes exhibit chromosome 1 rearrangements (23–25). The primary mechanism for 1q amplification in MM has been described as a process called “jumping translocation”, where the 1q arm translocates to several recipient chromosomes and the 1q copy number can increase over time (26–29). This syndrome frequently involves 1q12 PCH, which seems to acquire self-propagating mobile properties that drives continuous duplication/deletion events. In most patients, this results in 1q copy numbers of 3 to 4, but in some patients a process called “1q12–21 breakage-fusion-bridge cycle amplifications” can generate ladders of 1q12–21 amplicons (30). 1q jumping translocations are also common in multiple other types of hematopoietic cancers and are also observed in solid cancers (31). The involved breakpoints seem to vary with 1q10–21 depending on the cancer type.

## The Role of Chromosome 1q Amplification in Cancer Development

It is evident that gain of chromosome 1q is a recurrent aberration in many types of cancer and is invariably associated with poor outcomes and disease recurrence (26, 32–39). The 1q arm host many well-known oncogenes such as *NRAS, JUN, MYCL, TAL1, BLYM, LCK*, of which the amplification may increase expression levels and thereby support tumorigenesis. However, a number of additional candidate genes have been identified that may also be of importance. Genes of 1q21–23 are of special interest since this region is frequently subject to local amplification (17, 40–43) and this more often appears in aggressive tumors with metastatic potential and resistance to chemotherapy (44–47). In a study of breast cancer, 1q21–23 genes were directly implicated in the phenotype of breast cancer cells. The region was amplified in 10% to 30% of primary tumors and 70% of recurring tumors and was associated with early relapse and resistance to chemotherapy (48). This phenotype was attributed to the *S100A* family genes, which was demonstrated to support oncogenic traits on breast cancer cells. This region includes several other potential oncogenes such as *ALC*, which is frequently amplified and overexpressed in hepatocellular carcinoma and increase tumorigenicity in mouse models (49) as well as *MCL-1*, which contributes to survival of multiple myeloma cells, and correlates with poor prognosis (50). Candidates such as *RAB25, NES, CRABP2, HDGF and NTRK1* among others remain less characterized (22, 51). Thus, many genes on 1q21–23 and 1q in general may be involved in tumorigenesis and most likely different genes may give selective advantages to different subsets of tumors. This may explain the frequent amplification of the 1q arm in cancer.

## Epigenetic Control of 1q12 Pericentromeric Heterochromatin Repression

**Figure 1:**
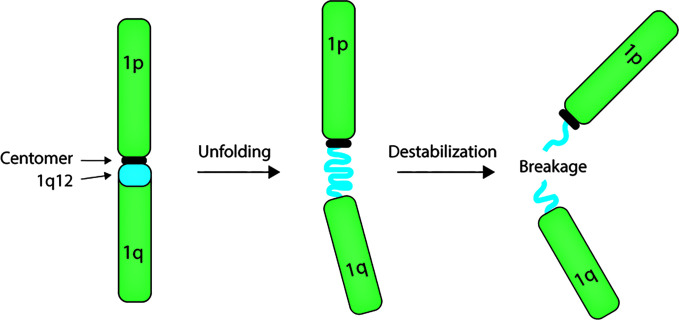
Model for 1q12 pericentromeric heterochromatin involvement in rearrangement of the 1q arm. The megabase 1q12 pericentromeric heterochromatin is composed of satellite II and III DNA repeats. In multiple types of cancer, this domain unfolds and becomes instable, leading to rearrangement and amplification of the 1q arm.

The mechanism of chromosome 1q amplification involving 1q12 PCH rearrangements seems to involve unfolding of the 1q12 PCH (**Figure 1**) (29, 52–57), which may result from decondensation of the chromatin structure. We have demonstrated that this unfolding implicates 1q12 PCH in the formation of chromatin bridges during mitosis and the formation of post-mitosis micronuclei (57), which are both clear signs of genomic instability and indicative of 1q12 instability. This destabilization of 1q12 PCH may arise from loss of epigenetic control of chromatin structure.

In most types of healthy cells, PCH is contained in a repressed state to maintain its stability. This is achieved by a specific epigenetic footprint, including H3K9me3, deposited by the SUV39H1/2 lysine methyltransferase. This mark recruits heterochromatin protein 1 (HP1) that interacts with other epigenetic factors to implement a repressive state, involving additional repressive marks such as DNA methylation and H4K20me2/3 (4, 58). Over the recent years, novel players in structural maintenance of PCH have been identified. This includes species of non-coding RNA transcribed from these regions, showing that PCH is not as transcriptional silent as previously anticipated. Interestingly, these ncRNAs have been demonstrated to be implicated in the repression of PCH by different mechanisms. For instance, PCH RNAs are processed by dicer and the resulting siRNAs target PCH to facilitate H3K9 methylation (59). Other studies have demonstrated that HP1 binding to PCH is RNA-dependent and involves long non-coding RNA. Importantly, in several studies core phenotypes of cancer cells have been attributed to overexpression of PCH satellite RNAs (59).

An important link between PCHs and aging has is also well established and may further tie PCH dysregulation to cancer development (60). Evidence from many different models of cellular senescence and organismal aging suggests that the aging process is associated with PCH loss. For instance, one of the core markers of heterochromatin, H3K9me3, is gradually reduced during aging and even though senescent cells display the formation of senescence-associated heterochromatin foci (SAHF) this is accompanied by a global loss of heterochromatin (61, 62). Similar results have emerged from premature aging syndromes. This includes Hutchinson–Gilford progeria (HGPS), which is associated with H3K9me3 and HP1 loss and is caused by inactivation of Lamin A (63, 64), an important factor of heterochromatin regulation at the nuclear lamina. The mechanisms underlying age-related epigenetic reprogramming of (pericentromeric) heterochromatin and their role in the aging process remain largely elusive, but this and its possible relationship with PCH dysregulation in cancer should be further investigated.

In the recent years, also Polycomb group (PcG) repressive complexes have been acknowledged as regulators of PCH silencing. There are at least two types of PcG complexes, PcG repressive complex 1 and 2 (PRC1 and PRC2), which have been detected in different variants with distinct compositions and functions (65). PRC1 specifically recognizes H3K27me3 catalyzed by PRC2 and has E3 ubiquitin ligase activity for H2A, while PRC2 specifically interacts with H2AK119ub produced by PRC1. Originally, PRC2 deposition on chromatin was believed to exclusively mediate PRC1 recruitment, but recent studies have revealed a more complex mechanism for PcG deposition. PcG proteins were traditionally specifically associated with facultative heterochromatin which was considered to be repressed by mechanisms distinct from those of PHC repression, but several studies have demonstrated that under some circumstances, PcG proteins can be found associated with PCH (66–70). The interplay between PcG and HP1 mediated silencing mechanisms in repression of PCH in homeostasis remains largely uncharacterized.

## The Role of DNA Methylation in Destabilization of 1q12 Pericentromeric Heterochromatin

**Figure 2:**
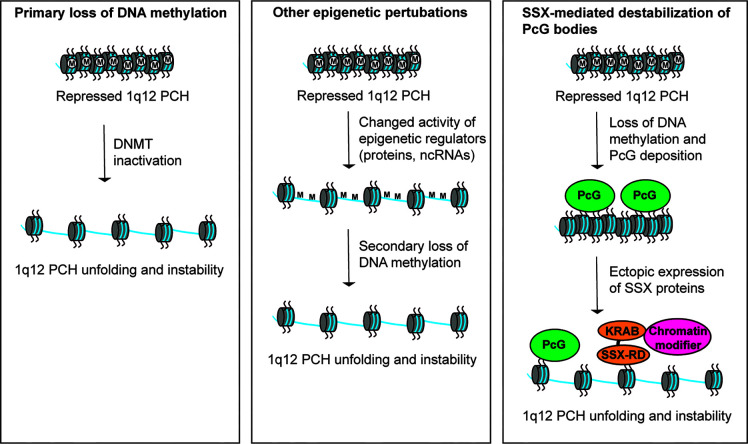
Different models for perturbation of 1q12 pericentromeric heterochromatin stability. (Left) Hypomethylation of satellite DNA in cancer cells leads to loss of epigenetic repression and subsequent unfolding and destabilization of 1q12 PCH. This may be caused by inactivation of DNA methyltransferases. (Middle) Deregulation of epigenetic mechanisms (other than DNA methylation) controlling the repression of 1q12 PCH may lead to unfolding and destabilization of 1q12 PCH associated with a secondary loss of DNA methylation. (Right) 1q12 pericentromeric DNA is epigenetically reprogrammed into PcG domains in response to loss of DNA methylation. These domains (i.e., PcG bodies) may be targeted by SSX proteins ectopically expressed in cancer cells leading to 1q12 PCH unfolding and instability.

Studies over the recent years have provided some mechanistic insight into the destabilization of 1q12 leading to genomic rearrangement of 1q. An important factor seems to be DNA hypomethylation, which is ubiquitously recognized in tumors and mainly affects CpG residues of repeated DNA sequences (71). Hypomethylation of PCH satellite DNA is a common event in cancer and may perturb normal control of chromatin structure (8, 55, 72–74). The first clue to the importance of DNA methylation in regulation of 1q12 PCH stability came from the disorder Immunodeficiency, Centromeric instability and Facial anomalies (ICF). This disease is characterized by decondensation and rearrangements of PCH regions, including 1q12, and has been demonstrated to be caused by inactivating mutations in the gene encoding DNA methyltransferase 3B (75, 76). Also in cancer, DNA methyltransferase genes are frequency deleted or mutated and their inactivation can cause genomic instability (77). In line with this, loss of DNA methylation has been associated with 1q12 PCH instability in cancer cells (**Figure 2**, left) (55, 78–80). For instance, hypomethylation of 1q12 was associated with 1q copy number gain in breast cancer (78) and a strong correlation between hypomethylated Sat2 sequences and 1q copy number gain with a 1q12 breakpoint was found in hepatocellular carcinoma (55). In the latter study, hypomethylation of Sat2 was also detected in normal tissues adjacent to the tumor in many patients, suggesting that this aberration occurs as an early event in the progression towards malignancy. Furthermore, 1q12 PCH unfolding and instability can be induced in various cell types by treatment with DNA methyltransferase inhibitors (72, 74, 81). Thus, loss of DNA methylation seems to be instrumental for 1q12 unfolding and destabilization in cancer cells, but causality between DNA methyltransferase inactivity and 1q rearrangement still remains to be demonstrated. Deregulation of other factors that affect DNA methylation or epigenetic regulation of heterochromatin compaction in general may also drive loss of structural maintenance and destabilization 1q12 PCH (**Figure 2**, middle). One example is the histone demethylase KDM4A, which causes rereplication and site-specific copy number gains of 1q12 and 1q21 (82).

## PcG Proteins in Epigenetic Regulation of 1q12 Pericentromeric Heterochromatin

We, and others, have recently demonstrated that 1q12 PCH undergoes epigenetic reprogramming by PcG proteins in premalignant and malignant lesions (74, 81). In many different types of cancer, PcG proteins can be found in relatively large nuclear aggregates referred to as PcG bodies. We have showed that PcG bodies are in fact nuclear subdomains in which PRC1 accumulates on the 1q12 PCH (74). In melanoma, these structures are present in up 80% of tumors with PcG expression and also frequently found in benign nevi, but not in any of the investigated normal tissues. This suggests that epigenetic reprogramming of 1q12 PCH is an early premalignant event in melanoma development and perhaps in other cancer diseases. Interestingly, the formation of PcG bodies correlated with loss of 1q12 satellite DNA methylation and a general reduction in total DNA methylation levels (74), suggesting that this change in the epigenetic profile of 1q12 PCH was initiated by cellular hypomethylation. This was supported by the induction of PcG bodies in primary melanocytes by treatment with DNA methyltransferase inhibitors. In this scenario, loss of DNA methylation and repressive factors such as HP1 and H3K9Me2/3 that in concert repress PCH in normal cells may act as nucleation sites for PRC1 binding. Indeed, PcG complexes have been demonstrated to bind to hypomethylated DNA (83–85). Interestingly, the epigenetic reprogramming of 1q12 PCH into PcG domains correlated with increased expression of 1q12 satellite RNA. Whether these RNA species were instrumental for the deposition of PRC1 complexes on 1q12 PCH remains to be determined. The features of the observed epigenetic reprogramming of 1q12 PCH (i.e. loss of DNA methylation and increased satellite transcription) were similar to those described in association with destabilization of 1q12. However, cancer cells with PcG bodies exhibited no unfolding of 1q12 PCH and no signs of genomic instability. With this in mind it can be speculated that PcG bodies are formed as a compensatory repressive mechanism to loss of DNA methylation-mediated repression. PcG bodies have also been suggested to work as molecular sponges to sequester PcG proteins thereby depleting them from other genomic sites (81).

## SSX-Mediated Destabilization of PcG-Repressed 1q12 Pericentromeric Heterochromatin

The formation of PcG bodies in premalignant and malignant cells is interesting in relation to the expression of SSX (synovial sarcoma, breakpoint X) proteins in cancer. This family consists of 9 highly identical members only expressed in pre-meiotic male germ cells of healthy individuals (86, 87). However, these proteins are also expressed in most types of cancer due to demethylation of their gene promoters (86, 88–91). A link between SSX molecules and PcG proteins in chromatin regulation has been demonstrated in multiple studies and SSX proteins target PcG bodies (57, 92–94). Importantly, we have recently shown that SSX proteins deplete PcG bodies in cancer cells and induce genomic instability (93, 95). Further studies demonstrated that SSX proteins promote the unfolding and derepression of 1q12 PCH during replication (57). In turn, this led to segregation abnormalities during anaphase and generation of genomic instability in the form of anaphase bridges and micronuclei (**Figure 2**, right). Depletion of PcG factors from cells with PcG bodies did not phenocopy SSX expression in these cells, suggesting that the structural modification of 1q12 PCH inflicted by SSX proteins was a direct effect of SSX binding to this chromatin domain rather than being caused by the depletion of PcG factors. These results implicate SSX proteins in destabilization of PcG repressed 1q12 PCH. Whether this is instrumental for the 1q12 rearrangements seen in solid and hematological cancers remains an important subject of investigation.

## Conclusion

Given the frequency of 1q rearrangements in cancer and the obvious contribution of 1q12 PCH it seems of high importance to understand the etiology and consequences of this genomic perturbation. Several questions remain unanswered in regard to the deregulation of 1q12 PCH. It is important to better understand the highly complex machinery maintaining epigenetic control of this domain, including the contribution of individual factors. For instance, the role on non-coding RNA species in stabilization and destabilization of 1q12 PHC (and other PHC domains) still remains largely elusive. Another subject where we have only scratched the surface is the surprising conversion of 1q12 PCH into PcG domains in premalignant and malignant cells. This may occur as a consequence of loss of DNA methylation, but it remains elusive what these cell types gain from this. Important clues may come from the involvement of PcG proteins in multiple facets of tumorigenesis or from the recent implication of PCH in senescence development (80, 96). Further understanding of other factors that may destabilize 1q12 PCH, such as KDM4A or SSX, will be equally important.

Attention should also be given to investigating the involvement of 1q amplifications in tumorigenesis. This chromosome arm contains multiple oncogenes, which may increase their expression through genetic amplification and contribute to acquisition of cancer hallmarks. However, only few of these genes have been investigated and much work remains on characterizing the role of individual genes in different cancer diseases. This will not be trivial since different genes may be important in different cancer types and several genes may work in concert to promote the development of cancer hallmarks. Furthermore, it must be emphasized that further progress in this research field will be driven by a coordinated understanding of the genetic, molecular and functional events that cooperate to support tumor development and progression. In this context, tumor heterogeneity and genetic mosaicism should be considered as important contributing factors. Thus, analysis of genetic aberrations and RNA/protein expression at the single-cell level will be highly important. While the road to a complete understanding of the role of instability of PCH domains in cancer remains challenging it may bring novel diagnostic, prognostic or therapeutic opportunities.

## Author Contributions

The author confirms being the sole contributor of this work and has approved it for publication.

## Funding

This work was supported by the Velux Foundation, the Danish Cancer Society (R146-A9213-16-S2), the Academy of Geriatric Cancer Research (AgeCare), the Novo Nordisk Foundation (NNF18OC0052303), the Danish Research Council for Independent Research (6108-00372A), the A.P Møller Foundation, Fabrikant Einar Willumsens Foundation, the Danish Cancer Research Foundation and Læge Sofus Carl Emil Friis og Hustru Olga Doris Friis foundation.

## Conflict of Interest

The author declares that the research was conducted in the absence of any commercial or financial relationships that could be construed as a potential conflict of interest.
